# Aged and induced-premature ovarian failure mouse models affect diestrus profile and ovarian features

**DOI:** 10.1371/journal.pone.0284887

**Published:** 2023-12-08

**Authors:** Ana Carolina Zucon Bacelar, Nataira Regina Momesso, Felipe Haddad Martim Pederro, Alaíde Gonçalves, Edilson Ervolino, Antonio Hernandes Chaves-Neto, Claudia Cristina Biguetti, Mariza Akemi Matsumoto

**Affiliations:** 1 Department of Diagnostics and Surgery, São Paulo State University—Unesp, Araçatuba, School of Dentistry, São Paulo, Brazil; 2 Department of Basic Sciences, São Paulo State University—Unesp, Araçatuba School of Dentistry, São Paulo, Brazil; 3 School of Podiatric Medicine, The University of Texas at Rio Grande Valley (UTRGV), Rio Grande Valley, Texas, United States of America; Université Clermont Auvergne - Faculté de Biologie, FRANCE

## Abstract

Sex hormones exert a wide influence on several systems of the human body, especially in women, who undergo intense changes in the trans and postmenopausal periods. Different experimental models are used to mimic these conditions; however, the impact on hormonal profile may be different. This study aimed to analyze and compare vaginal cytology of different post-estropausal mice models, along with their microscopical ovarian features. Forty-six C57BL/6J female mice with the ages of 4, 6 and 18 months at the beginning of the experiment, weighing about 25–28 grams, constituted five groups: NC–(negative control) animals with no treatment, OVX-SHAM—sham ovariectomized, OVX–ovariectomized, VCD–medicated with 160 mg/kg/day of 4-vinylcyclohexene diepoxide via IP for 20 consecutive days, and Aged–senescent mice under physiological estropause. Euthanasia was performed at different periods for the removal of the ovaries, and after diestrus was confirmed by vaginal cytology for 10 consecutive days. For daily vaginal cytology, morphological and histomorphometric microscopic analyzes were performed. Aged mice presented significant increased neutrophils when compared to VCD group, as well as increased cornified epithelial cells when compared to OVX mice, and also increased nucleated epithelial cells when compared to VCD and OVX. NC and OVX-SHAM ovaries presented innumerous follicles at different stages of development, while VCD showed marked follicular atresia, depleted of primordial or developing follicles and a predominance of interstitial cells. The ovaries of aged mice were predominantly constituted by corpus luteum degenerated into corpus albicans, with rare antral follicles. All analyzed models led to different permanent diestrus profiles caused by each model, as indicated by ovarian features. This should be carefully considered when choosing a post-estropausal experimental model, in order to better correlate this challenging phase of female’s life with physiological/pathological conditions.

## Introduction

Sex steroids are directly related to humans’ health since they influence overall physiology, affecting not only a number of systems as immune and skeletal, but also our emotional behavior [1, 2]. This wide rage influence of sex hormones is the fuel for the development of interesting experimentation in order to explore hormonal changes using *in vivo* models [3–5]. Among the several responses sex steroids disturbances can cause in an individual, undoubtedly women are specially affected, mainly at the end of the reproductive phase, due to ovarian failure and the drastic decrease of estrogens and progesterone, marking perimenopause and menopause events [6].

Excluding non-human primate models, rodents are valuable animals for experimental study of the effects of female hormone deficiency due to their easy handling, short lifespan, and well described reproductive and aging profiles [7]. However, differently from women that initiate their reproductive phase marked by the menstrual cycle, female rodents do note menstruate, but present an estrous cycle when their uterine lining is resorbed instead of eliminated [8]. Four phases comprise the estrous cycle in a period of 4–5 days: proestrus, estrus, metestrus, and diestrus, controlled by steroid hormones and that can be identified via vaginal cytology [9]. Estrus is the stage in which females are more prone to copulate, with high levels of progesterone (P4) at the end of the cycle, and characterized by the abundance of cornified cells in the cytology. In metestrus, 17β-estradiol returns to basal levels, and increased leukocytes accompanied by a decrease in cornified cells are observed. Diestrus represents the resting phase of reproductive cycle, and cell components are strongly diminished, before proestrus phase begins with an increase of 17β-estradiol followed by a luteinizing hormone (LH) surge that induces ovulation, resulting in a predominance of nucleated epithelial cells [10]. Estrous cycle cytology is a result of the presence of estrogen receptor (ESR1) in the epithelial cells of the female reproductive tract, which regulates the differentiation of vaginal mucosa [11]. Also, the levels of ESR1 ligand modulate inflammatory genes and antimicrobial proteins [12]. At the end of reproductive phase, female rats and mice also experience oscillatory hormonal and ovarian cycling, resulting in a persistent diestrus condition [9].

Undoubtedly, the surgically induced premature ovarian failure (POF) model by ovariectomy (OVX) is the most executed technique in order to mimic menopause condition [13]; however, the abrupt interruption of the main source of steroids in females caused by this procedure does not appropriately reflect the hormonal cycle of this phase, since it does not allow the natural transition from reproductive to non-reproductive life stage events to occur [14]. It is of relevance since in a natural senescent human condition, menopause is a consequence of a gradual failure of ovarian function caused by the depletion of the small preantral follicles (primordial and primary ovarian follicles). This process can last up to ten years [15], resulting in irregular menstrual cycle and causing important discomforts as hot flashes, along with physical and cognitive alterations [16, 17], by the disruption of hypothalamic-pituitary-gonadal (HPG) axis and its influence in neuroendocrine feedback as women age [18, 19]. Since these events are chronological and aged-related, they are better reproduced using intact-ovary rodent models [20]. In addition, OVX animals are subjected to a number of systemic conditions, as high adiposity and insulin resistance that predispose them to metabolic and cardiovascular diseases [21], decreased performance regarding short and long-term memory due to disturbance of hippocampus neurons [22], and increased inflammatory responses [23].

In order to better reproduce human perimenopausal to post-menopausal progression, Mayer et al. [24] developed a chemically induced model of POF using the industrial chemical 4-vinylcyclohexene diepoxide (VCD) in intact- ovary mice model. VCD is a diepoxide metabolite of 4-vinylcyclohexene (VCH), a commercial chemical used as intermediate and reactive diluent for diepoxide and epoxy resins [25]. Although previous study has attested the ovotoxicity of VCH for small preantral follicles in rats and mice, it showed less efficiency in rats [26]. Daily repeated treatment with VCD selectively eliminates small ovarian follicles by affecting important pathways involved in apoptosis as Bax/Bcl2 pathway [27], in the communication between the oocyte and surrounding granulosa cells, necessary for oocyte survival as the KIT/KITLG signaling pathway [28, 29], and also the Rictor/mTORC pathway [30]. This gradual depletion results in the retention of residual ovarian tissues, with the presence of stromal and interstitial tissues that continue to secret androgens [31], that results in similar hormone profiles with reduction of 17β-estradiol and increase of FSH [2, 8, 24, 32], mimicking human condition and that might be involved in the associated menopause diseases [24].

Considering the outcomes of OVX and VCD POF models on female sex hormones, and the influence of 17β-estradiol on vaginal epithelial lining, one can question if this could not impact in estrous cycling profile of rodent models, especially when persistent diestrus is desired to confirm the end of reproductive phase of the animal. In order to answer this questioning, the aim of the present study was to compare estrous cycle of young C57Bl/6J female mice OVX and VCD POF-induced, and matched senescent animals, along with ovarian histological aspects, in order to guide researchers to better correlate and explore human post-menopausal phase to their specific investigation based on each of these models.

## Methods

### Animals and ethical approval

In order to match the ages of the young mice, and also to wait for the experimental procedures to induce POF, animals of different ages at the beginning of the experiment were used. Forty-six multiparous female C57BL/6J mice were included, from which 10 were 18 months-old (aged), with mean weigh of 28 grams, 10 were 6-months-old, and 30 were 4 months-old (young), mean weight of 25 grams, supplied from Central Animal Facility for Special Mice of the School of Medicine of Ribeirão Preto–University of São Paulo, Brazil (CCCE-FMRP-USP), and housed in the Central Animal Facility of São Paulo State University, School of Dentistry, Araçatuba, São Paulo, Brazil, under the written approval of Institutional Ethical Committee on Animal Use (Protocol CEUA-FOA #00910/2019) that stated the experimental protocol of the project was in accordance with the Ethical Principles of Animal Experimentation, and following normative regulations of the National Council for the Control of Animal Experimentation (CONCEA) in Brazil. The animals were maintained in appropriate cages with a maximum of 5 mice in each one, under controlled temperature (22 ± 2oC) and light cycle (12 h dark/light) room, with no restriction of water and food intake.

### Experimental groups

The female mice were divided in five groups according to the age and treatment, as follows: NC (negative control)–no treatment (n = 6; 6 months-old), OVX-SHAM–fictitious ovariectomy (OVX) surgery (n = 10; 4 months-old), OVX–OVX (n = 10; 4 months-old), VCD—daily treated with 160mg/Kg of 4-vinylcyclohexene diepoxide (n = 10, 4 months-old) and Aged no treatment (n = 10; 18 months-old).

### OVX and OVX-SHAM procedures

Two calibrated surgeons (ACZB and NRM) performed all the surgical procedures. First, the animals were individually weighted immediately before the surgery for the correct dose of the anesthetic drugs. The mice were anesthetized with IM injection of 1% ketamine chloride (80mg/Kg) (Dopalen, Agribrans do Brasil LTDA, SP, Brazil), in association with 2% xylazine chloride (15mg/Kg) (Anasedan, Agribrans do Brasil LTDA, SP, Brazil) and OVX procedure was performed as previously described [33]. Briefly, after trichotomy and antisepsis with 1% polyvinylpyrrolidone of the abdominal region, a 5 mm incision was performed in the dorsolateral area of the abdomen between the last rib and the hip. After localizing the ovaries, the ovarian fat was carefully pulled throughout the incision to permit the exposure of the oviduct. A ligature was made using a sterile nylon suture in order to delimit the area to be removed and to avoid bleeding after the removal of the ovaries. Using a small surgical scissor, the ovaries were separated from the oviduct, which was put back in the abdominal cavity. The incision was closed using 5.0 nylon suture (S1 Fig). The animals were kept in individual cages and under observation until they fully recovered from the surgery. After 24 hours, they were back together with the same animals they were before, and that underwent the same procedure.

OVX-SHAM procedure followed the same surgical steps of OVX, except by the excision of the ovaries, which were exposed and returned to the original place.

### VCD treatment

VCD (Sigma-Aldrich, St. Louis, USA) was diluted in sterile 0.9% saline solution and administered to the animals during 20 consecutive days via IP, in the dose of 160 mg/Kg/day [32].

### Vaginal cytology for qualitative and quantitative analysis

The estrous cycle of OVX, VCD, and Aged mice was monitored daily by toluidine blue-stained vaginal cytology for 10 consecutive days, as described elsewhere [9], with OVX-SHAM and OVX animals starting from days 20 to 29 after surgeries, therefore, at the end of the vaginal cytology, they were about 5 months old. The VCD group had the collections carried out from days 52 to 61 after the first dose of VCD, presenting 6 months at the end of the cytology and aged mice when they completed 18 months, the collections were carried out from days 0 to 9.

OVX, VCD and Aged periods were determined in order to achieve ovarian failure. Briefly, vaginal lavage was performed carefully introducing a pipette with 50μl of 0.9% saline solution in the vaginal canal of the animals, approximately 1–2 mm deep. Two to three gently washes were made in order to guarantee a satisfactory sample. The lavage was put on a histological slide and left to air dry, to be stained with toluidine blue for one minute. After, the slides were washed in distilled water for one minute. Cytology was analyzed under light microscopy and diestrus phase was considered when a predominance of neutrophils, moderate nucleated epithelial cells, and eventual anucleated keratinized epithelial cells (cornified epithelial cells) were observed. Histological slides of each vaginal lavage were analyzed under light microscopy qualitatively and quantitatively. For histomorphometry, photomicrographs of the slides were obtained under 40x magnification and a grid with 266 intersection points was used to quantify the cells types using ImageJ software (NIH and LOCI, University of Wisconsin). The obtained data were analyzed by Kruskal-Wallis followed by Dunn’s test, considering p<0.05.

### Ovaries removal for qualitative and quantitative analysis

The animals were euthanized under anesthetic overdose and the ovaries collected. Euthanasia was performed in different periods, considering each model and after diestrus was confirmed, as follows: NC and Aged mice after 21 days, OVX and OVX-SHAM after 27 days, and VCD after 46 days. OVX mice were also euthanized despite the absence of the ovaries. Immediately after the removal, the ovaries were immersed in buffered 10% formalin and after 48 hours, washed in running water for 12 hours and were prepared to be stained with hematoxylin and eosin (HE). Qualitative analysis considered the aspects of medullar and cortical areas, along with the presence/absence of the ovarian follicles.

For quantitative analysis, images of the ovaries at 4x magnification were submitted to ImageJ software (NIH and LOCI, University of Wisconsin) analysis. Initially, the images were standardized using a scale bar, to carry out the measurements. For area measurement, the Freehand selections tool was used, contouring the entire length of the border of the ovaries to obtain the measurement of the area in μm^2^. For the measurement of ovaries diameter, a straight line was traced considering the largest diameter of the ovaries using the Freehand line tool, from wich the diameter was calculated (μm). Obtained data underwent statistical analysis under One-way ANOVA followed by Tukey, considering p<0.05.

## Results

### Vaginal cytology revealed different cells proportions during permanent diestrus depending on post-estropausal model

**Fig 1:**
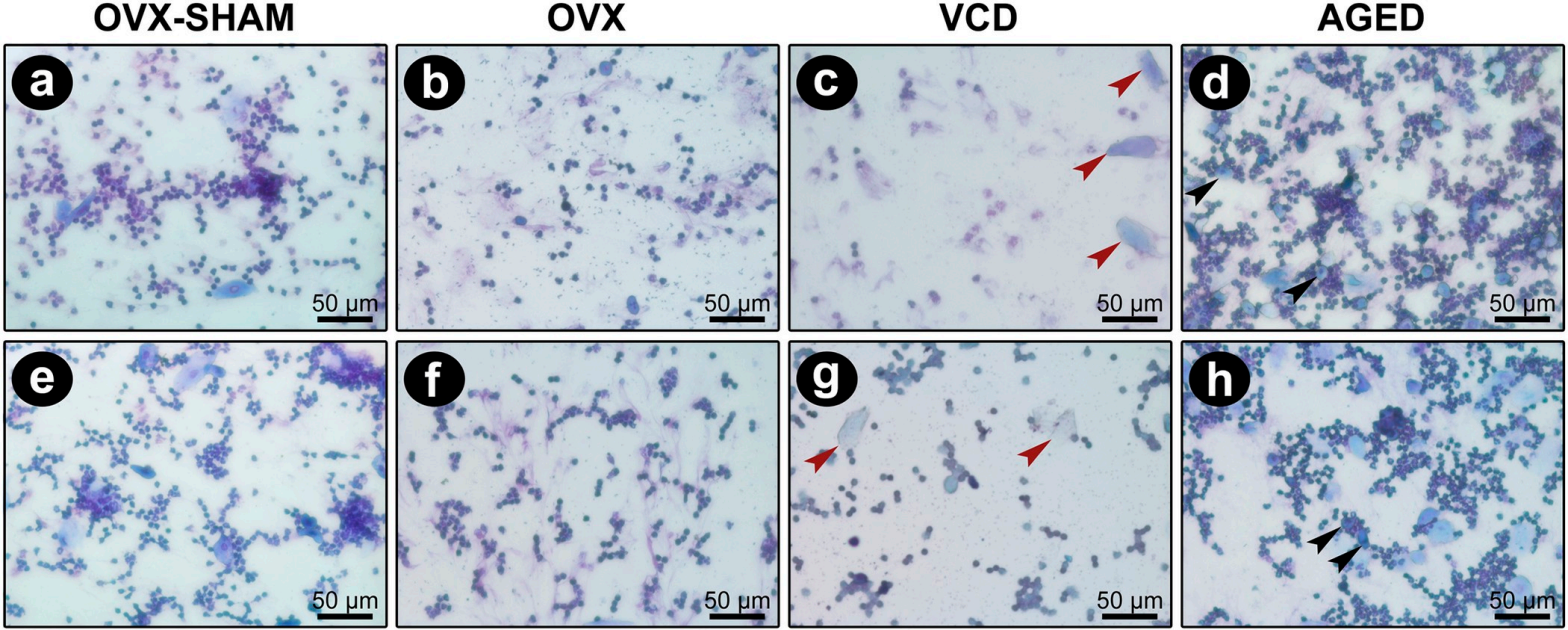
Cytological aspects of the vaginal lavage of OVX-SHAM, OVX, VCD, and Aged groups. **a, b)** OVX-SHAM exhibiting intense neutrophil infiltrate (green arrows) with eventual nucleated epithelial cells (black arrow); **c, d)** OVX group presenting moderate neutrophil infiltration (green arrows); **e, f)** VCD presenting cornified squamous epithelial cells (red arrows) amongst the neutrophils; **g, h)** Aged mice showing intense neutrophil infiltration along with numerous nucleated epithelial cells (black arrows). (Toluidine blue, 40x magnification).

Vaginal cytology confirmed the permanent diestrus phase in the animals of OVX, VCD, and Aged groups. Interestingly, although the cell types that characterized diestrus were observed, proportion was clearly different among the groups (Fig 1). Statistical analysis was performed comparing the experimental groups OVX, VCD, and Aged groups, and detected significant decreased neutrophils in VCD (12.6±9.813) in comparison to Aged mice (63.2±25.72). However, nucleated epithelial cells were significant increase in Aged animals (7.2±2.775) when compared to VCD (0.8±1.304) and OVX (0.00±0.00), and cornified epithelial cells were significantly increased in Aged mice (10±3.742) in comparison with OVX (0.4±0.5477) (Table 1, S2 Fig).

**Table 1:** Mean±standard deviation obtained from the quantification of histological parameters.

	VCD	OVX	Aged
**Neutrophils**	12.6±9.813^a^	39±9.644^b^	63.2±25.72^b^
**Cornified epithelial Cells**	6±1.871^ab^	0.4±0.5477^a^	10±3.742^b^
**Nucleated epithelial cells**	0.8±1.304^a^	0.00±0.00^a^	7.2±2.775^b^

Different letters indicate statistical differences among the groups, considering each histological parameter (p<0.05).

### Different post-estropausal models exhibited distinct ovarian features

Evident differences could be observed among the ovaries after their removal, mainly in relation to their sizes. Considering the microscopic aspects, the NC and OVX-SHAM groups presented similar ovarian characteristics with several ovarian follicles in different stages of development, from primary follicles to ovulatory follicles. Primordial and primary follicles presenting single-layer cubic granulosa cells were predominantly concentrated in the cortical area. Secondary and antral follicles were also seen, along with some eventual follicles in atresia.

**Fig 2:**
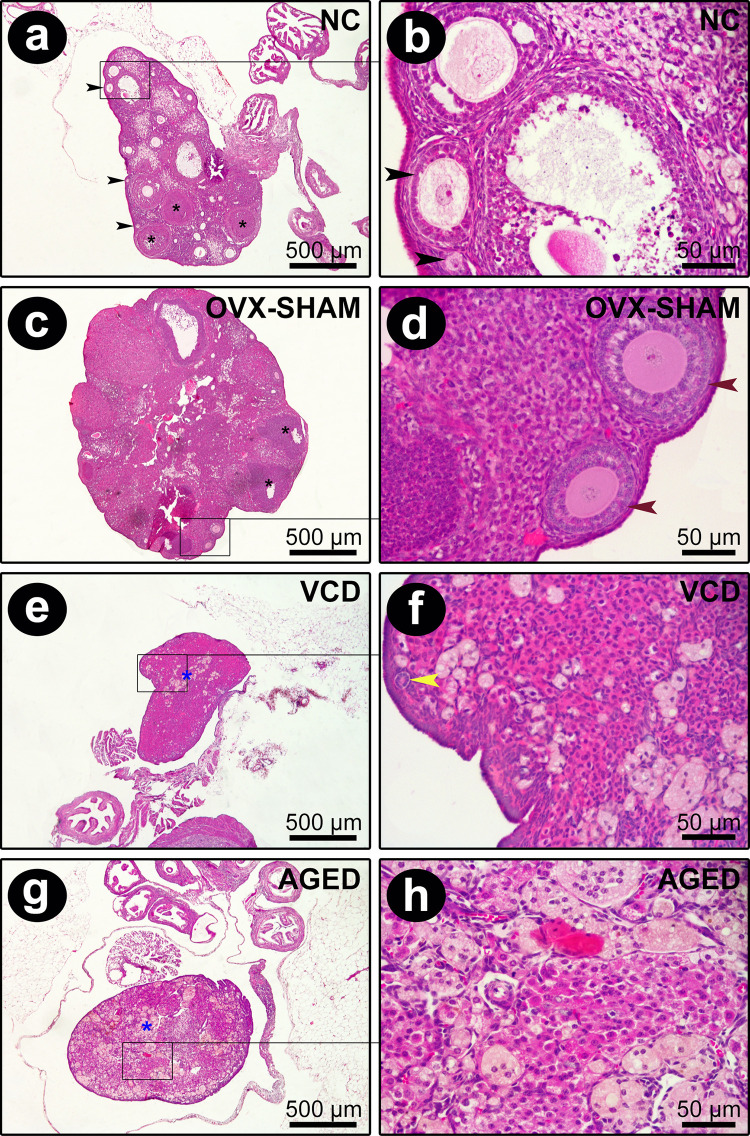
Histological aspects of the ovaries collected from each group. **a, b)** In NC, primary and secondary follicles are concentrated in the cortical region (black arrows). **c, d)** OVX-SHAM ovaries presented unilaminar primordial and primary follicles. It is also observed the presence of multilaminar primary follicle (red arrows) and collagen scars (blue asterisk). **e, f)** VCD ovaries presented no growing ovarian follicles; however, it is possible to note atresia-stage follicle in the peripheral region of the ovarian cortex (yellow arrows) and corpus luteum, which subsequently involutes and is replaced by a collagen fiber scar and becoming the corpus albicans (*). **g, h)** The ovaries collected from Aged group are mostly constituted by areas of the corpus luteum that become corpus albicans (blue asterisk) (HE; 4x and 40x magnifications).

**Fig 3:**
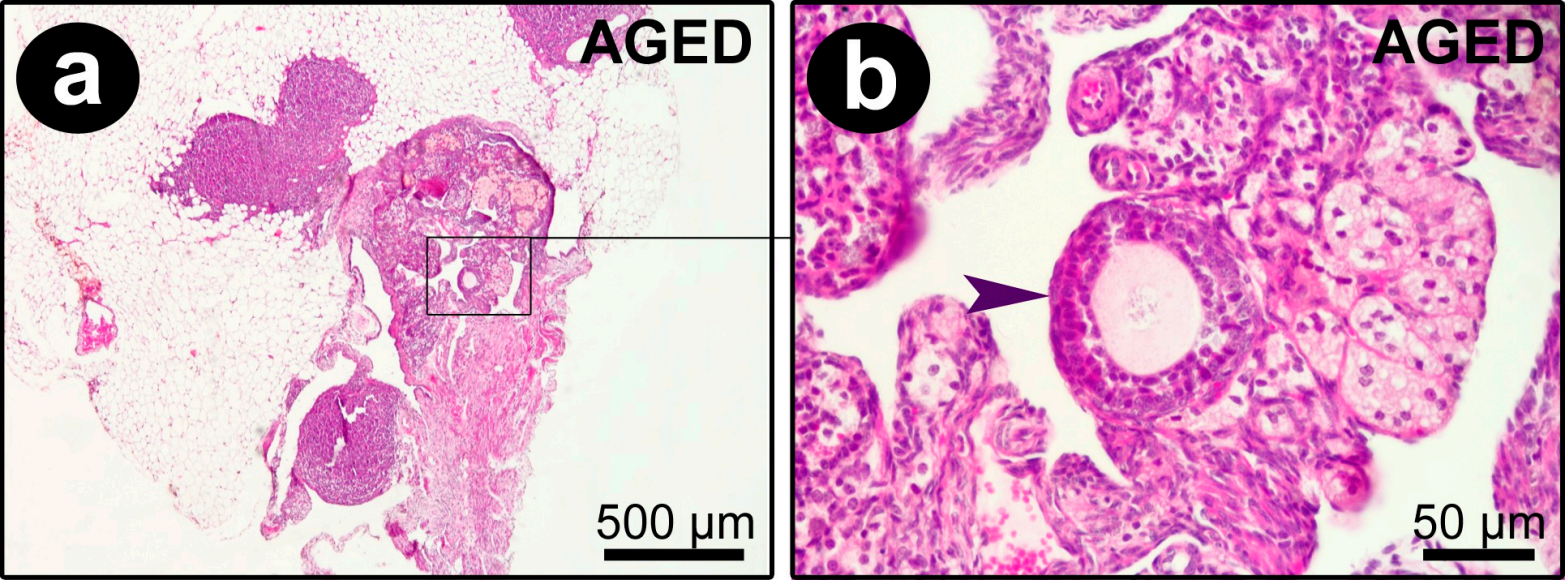
Ovary collected from Aged group. An antral follicle was detected in one of the aged mice (arrow) (HE; 4x and 40x magnifications).

When analyzing Aged and VCD-treated mice, it was clear that aging and VCD drastically affect the ovaries when compared to NC mice. While NC exihibited large ovaries with several ovarian follicles at different stages of development, as previously described, both VCD and Aged mice, but mainly VCD, showed marked follicular atresia. Ovaries from VCD animals were seen to be depleted of primordial or developing ovarian follicles, and follicular atresia was evident in the ovarian cortex. There was a predominance of interstitial cells, permeated by focal corpus albicans. The ovaries of the Aged mice consisted predominantly of degenerating corpus luteum into corpus albicans, although rare follicular atresia could be observed (Fig 2). Interestingly, an antral follicle was detected in one of the aged mice (Fig 3).

**Fig 4:**
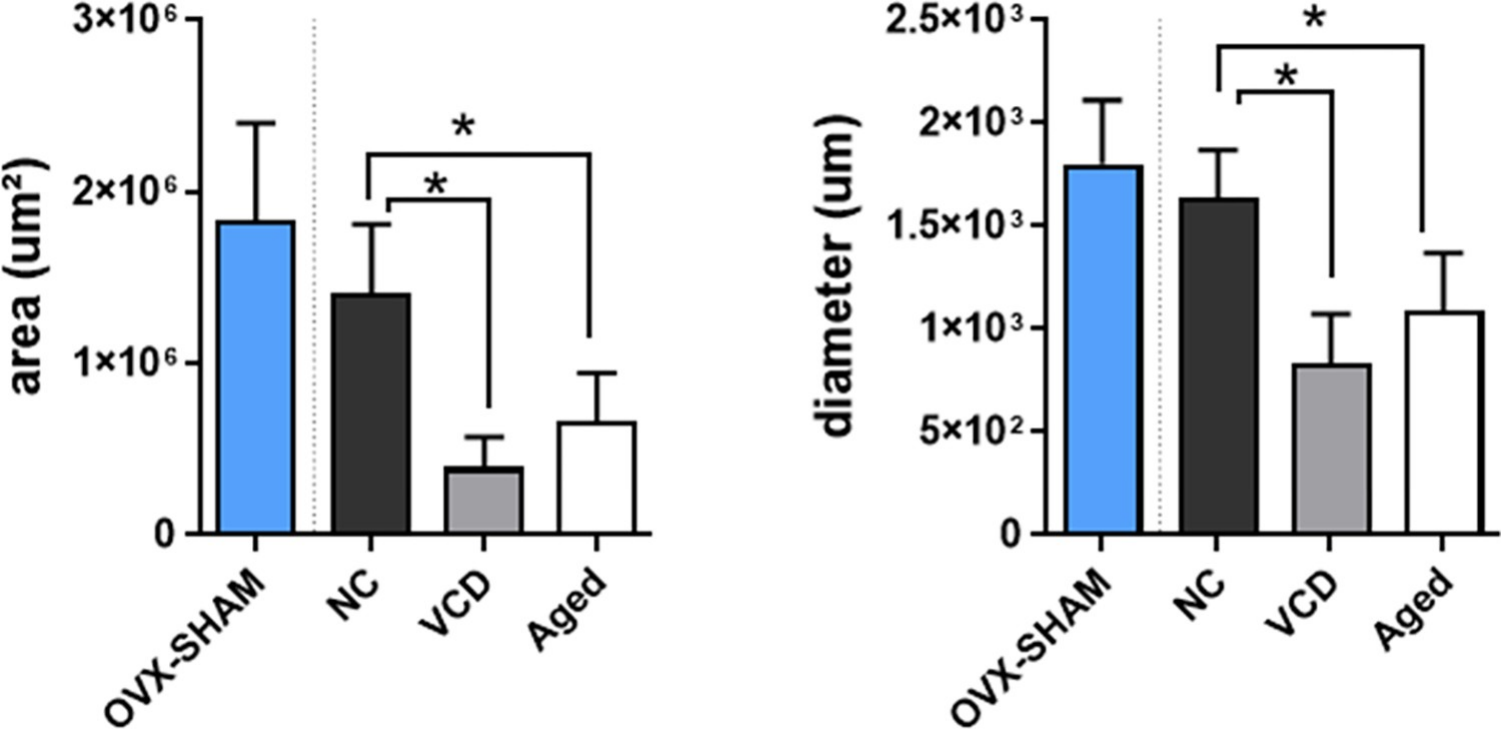
Quantitative analysis of the area and diameter of the ovaries of the OVX-SHAM, Aged, VCD and OVX groups. It was detected significantly reduction in the area and diameter of the VCD and Aged ovaries in comparison with the NC’s. OVX-SHAM group was not included in the statistics, but it can be observed that the SHAM procedure does not interfered in the dimensions of the ovaries. The data obtained were analyzed by the One-way ANOVA test followed by Tukey considering p<0.05.

From the quantitative analysis of the ovaries it was detected that VCD and Aged mice presented significantly reduced ovarian area and diameter when compared to NC (Fig 4).

## Discussion

The present investigation confirmed that chemically induced POF model in mice led to different outcomes when compared to natural senescence condition that may interfere in translational investigation, attested by ovarian features that suggested the hormonal status of the animals in each condition. Following this reasoning, vaginal cytology also showed distinct profile since vaginal epithelium, along with female reproductive tract, are directly dependent on estrogen by the presence of the ESR1, in order to regulate their differentiation [6]. However, important differences in gonadal hormone levels between rodents and humans have to be carefully considered in the post-estropausal/menopausal stage [9].

With the disruption of HPG axis and its influence in neuroendocrine feedback as women age, the decrease of GnRH by GnRH neurons in the hypothalamus weakens the signaling of the pituitary gland to synthesize the gonadotropins FSH and LH that are responsible for ovarian follicles to mature, resulting in low, or even undetectable levels of 17β-estradiol and progesterone, and high levels of the gonadotropins [34]. On the other hand, there are evidences that rodents can exhibit various stages of reproductive aging, and usually maintain moderate levels of circulating 17β-estradiol, despite HGP deregulation. In rodents, the beginning of irregular estrous cycle can manifest from 9–12 months of age, caused by hormonal fluctuations; however, they remain in peri-estropausal and not in a real post-menopausal, when compared to women [28].

Most of the women experience gradual decrease of ovarian function during years, in which the majority of the primordial follicles undergo atresia accumulating residual ovarian tissue composed by apoptotic granulosa cells and oocytes, becoming interstitial cells [6]. It is suggested that these cells contribute to androgen production in the climacteric ovary, and excessive androgen may be associated to some post-menopause diseases [24]. In this aspect, surgically POF induced by OVX in mice model may be contraindicated, since post-estropausal occurs immediately after the ovaries are removed, drastically ceasing the production of ovarian hormones and impeding peri-estropausal to occur [14]. Additionally, altered levels of estrogen ligand modulate the number of inflammatory genes and antimicrobial proteins [10].

Basically, OVX vaginal cytology was composed by neutrophils and mucous filaments, with less nucleated and cornified epithelial cells if compared to Aged and OVX-SHAM mice, reflecting the absence of epithelium activity controlled by estrogen. On the other hand, interstitial cells predominantly composed VCD ovaries, which were permeated by focal areas of corpus luteum. In turn, aged ovaries were full of corpus luteum developing to corpus albicans, and one antral follicle in one of the analyzed ovaries was detected. Thus, permanent diestrus of VCD mice was characterized by significant decreased neutrophils, and nucleated epithelial cells when compared to the aged mice. No differences were detected considering cornified cells. These cytology profiles may indicate the irregular hormone release by aged mice, different from what was observed in VCD’s. VCD close reproduces human perimenopausal to post-menopausal transition in women, since the period between the depletion of the small follicles (VCD target follicles), and ovarian collapse is similar to perimenopausal phase [32].

Follicle atresia clearly impacted in ovarian dimensions, as observed from the ovarian quantitative analysis, revealed by VCD and Aged groups. Both groups exhibited reduced ovarian area and diameter when compared with the NC animals. Significant reduced ovarian weight of VCD-treated mice has been previously reported when comparing with control animals [24].

Different protocols can be found in literature regarding the length and dosage of VCD for mice model. Mayer et al. (2004) [24] compared the effects of different VCD (80 mg/Kg– 1dose/day; 80 mg/Kg, 2 doses/day; 160 mg/Kg, 1 dose day; 249 mg/Kg, 1dose/day; 80 mg/Kg 3 doses/day; and 160 mg/Kg, 2 doses/day) and found that the 160 mg/kg, 1 dose/day for 15 consecutive days was the optimum dosage amongst the others, since it accelerated the loss of primary follicles without affecting the larger follicles or other tissues. After 37 days from the treatment, it was observed that there were no primordial or primary follicles left. However, we opted to follow Lohff et al. (2006) [32] methodology, which compared the effectiveness of 10 *vs* 20 consecutive days of VCD treatment, and observed that after 10 days from the onset of the 20-day treatment, all primordial and primary follicles were depleted and after 43–63 days ovarian failure was attested, while 10-day treatment presented depleted primordial and primary follicles after 104–166 days. In fact, after 62 days of VCD-treatment onset, no primordial or primary follicles were observed in the present study.

The results about hormonal levels from previous important investigations in mice POF models [2, 8, 24, 35] was considered, and it was not performed in the present study taking into account the 3R’s procedure [36], since this analysis would demand a significant increase in the quantity of animals. Nonetheless, it has been reported that hormone profile and the levels of gonadotropins after VCD administration in animal models are also quite similar to those of post-menopause women with intact ovaries [2, 8, 24, 32, 37]. The quantification of antral follicles in clinical researches is frequently used as a biomarker to attest the capacity of reproduction in the context of fertility and the transition to menopause, since it can be more accurate than serum levels of gonadotropin and gonadal hormone, considering that they erratically fluctuate in human menopause transition [8]. With VCD model it is possible to correlate the number of ovarian follicles and the reproductive senescence in rodents, and it also facilitates the comprehension about the impact of the post-estropausal intact ovarian tissue and the continuous androgen secretion and the relation with other circulating steroids produced by extra-ovarian tissues [8, 28]. In addition, although VCD administration leads to systemic distribution of the drug, no toxicity to other organs but to small ovarian follicles has been reported [38, 39].

## Conclusion

Physiological post-estropausal, and surgically and chemically induced POF led to different permanent diestrus profiles caused by each model, as indicated by ovarian features. This should be carefully considered when choosing a post-estropausal experimental model, in order to better correlate this challenging phase of female’s life with physiological/pathological conditions.

## Supporting information

S1 Fig OVX procedure. **a)** A dermal dorsolateral incision was made and a surgical access was performed to reach the ovaries. **b)** ovarian fat been pulled out to be ligate, **c)** removed ovaries. **d)** sutured incision.
(TIF)

S2 Fig Quantitative analysis of neutrophils, nucleated and cornified squamous epithelial cells of Aged, VCD, and OVX groups. The obtained data were analyzed by Kruskal-Wallis followed by Dunn’s test considering p<0.05. Asterisks indicate significant differences between the groups.
(TIF)

S1 Data (DOCX)
