# Supplementary figures and images for: Aged and induced-premature ovarian failure mouse models affect diestrus profile and ovarian features
Source: PLoS One. 2023 Dec 8;18(12):e0284887. doi: 10.1371/journal.pone.0284887 (PMC10707698; doi:10.1371/journal.pone.0284887)

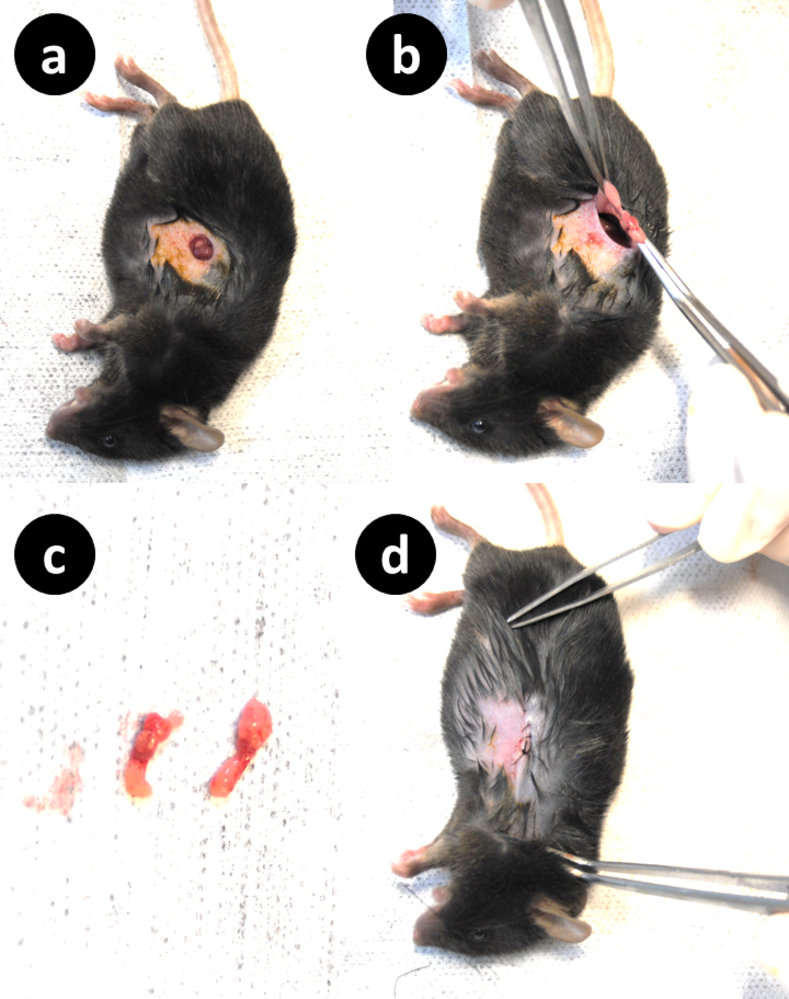

Supplement: S1 Fig — a) A dermal dorsolateral incision was made and a surgical access was performed to reach the ovaries. b) ovarian fat been pulled out to be ligate, c) removed ovaries. d) sutured incision. (TIF) [file pone.0284887.s001.tif]

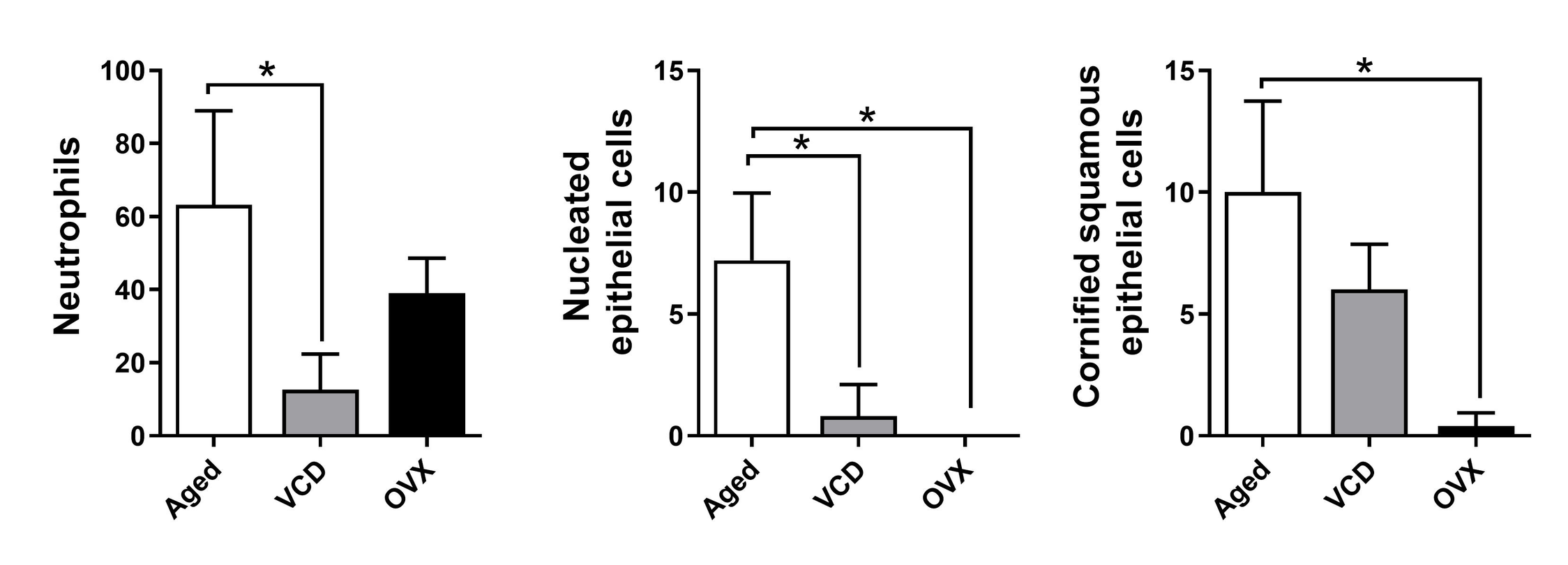

Supplement: S2 Fig — The obtained data were analyzed by Kruskal-Wallis followed by Dunn’s test considering p<0.05. Asterisks indicate significant differences between the groups. (TIF) [file pone.0284887.s002.tif]
