# Supplementary material for: Aged and induced-premature ovarian failure mouse models affect diestrus profile and ovarian features
Source: PLoS One. 2023 Dec 8;18(12):e0284887. doi: 10.1371/journal.pone.0284887 (PMC10707698; doi:10.1371/journal.pone.0284887)
Supplement: S1 Data — (DOCX) [file pone.0284887.s003.docx]

**SUPPLEMENTARY DATA**


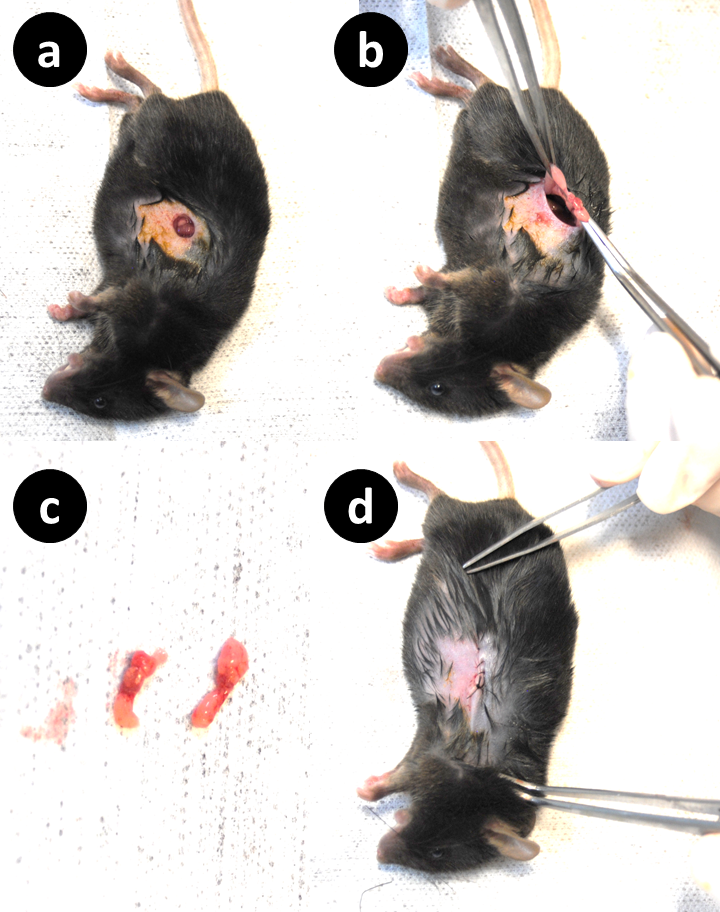


**Supplementary Fig 1.** OVX procedure. **a)** A dermal dorsolateral incision was made and a surgical access was performed to reach the ovaries. **b)** ovarian fat been pulled out to be ligate, **c)** removed ovaries. **d)** sutured incision.


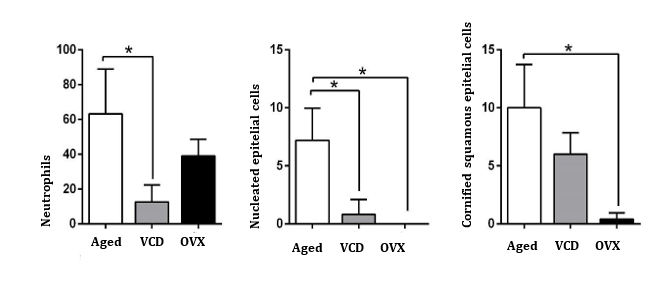


**Supplementary Fig 2.** **Quantitative analysis of neutrophils, nucleated and cornified squamous epitelial cells of Aged, VCD, and OVX groups.** The obtained data were analyzed by Kruskal-Wallis followed by Dunn’s test considering p<0.05. Asterisks indicate significant diferences between the groups.
